# Oncological safety of immediate breast reconstruction with skin- or nipple-sparing mastectomy: the value of tumor-to-dermis distance measured by preoperative ultrasonography

**DOI:** 10.1186/s12957-021-02185-7

**Published:** 2021-03-12

**Authors:** Takaaki Fujii, Yuko Nakazawa, Misato Ogino, Sayaka Obayashi, Reina Yajima, Chikako Honda, Hideharu Nakamura, Takaya Makiguchi, Ken Shirabe

**Affiliations:** 1 Division of Breast and Endocrine Surgery, Graduate School of Medicine, Gunma University, 3-39-22 Showa-machi, Maebashi, 371-8511 Gunma Japan; 2 Department of General Surgical Science, Graduate School of Medicine, Gunma University, 3-39-22 Showa-machi, Maebashi, Gunma 371-8511 Japan; 3 Department of Oral and Maxillofacial Surgery, and Plastic Surgery, Graduate School of Medicine, Gunma University, 3-39-22 Showa-machi, Maebashi, 371-9511 Gunma Japan

**Keywords:** Immediate breast reconstruction, SSM, NSM, Local recurrence, Ultrasonography, Breast cancer

## Abstract

**Background:**

Immediate breast reconstruction with skin-sparing (SSM) or nipple-sparing mastectomy (NSM) has become a common procedure. In this study, we evaluated the distance between breast tumor and skin in a series of patients undergoing IBR as it relates to oncologic safety, namely, the incidence of recurrence.

**Methods:**

The distance of the tumor to the dermis, rather than the outer layer of skin, was the key parameter of our preoperative ultrasound measurements. Our data set comprised the cases of 171 patients and 181 breasts with breast cancer that had undergone two-stage breast reconstruction by expander. The median age of the patients was 47 years (25–75 years). The overall median follow-up period was 47.1 months (8.8–125.3 months). Eighty-five breasts underwent IBR with SSM/NSM; the others underwent conventional mastectomy.

**Results:**

Among the total of 181 reconstructed breast mounds, the locoregional recurrence rate was 1.1% (2 breasts) with no cases of skin flap recurrence or skin flap necrosis. The tumor-to-dermis distance of cases with skin preservation (NSM/SSM) was significantly less than that of cases with conventional mastectomy (3.8 ± 2.7 mm vs 5.2 ± 2.4 mm). In cases with invasive carcinoma, all cases whose tumor-to-dermis distance was less than 2 mm underwent resection of the skin immediately overlying the tumor.

**Conclusions:**

Our results suggested that a 2-mm distance between the dermis and tumor on ultrasound evaluation is sufficient for the use of this tissue as a skin flap in SSM/NSM procedures. Our study indicated that immediate breast reconstruction with SSM/NSM can be an oncologically safe surgical option for breast cancer. However, we recommend that resection of the skin overlying the tumor be performed in cases with invasive breast cancer in which the tumor-to-dermis distance is less than 2 mm.

**Trial registration:**

Patients in this study were retrospectively registered. This study design was approved by our Clinical Ethics Committee (No 1297) (http://ciru.dept.showa.gunma-u.ac.jp/guidance/storage-sample/list.html).

## Background

In breast surgery, cosmetic results are often very important to patients. With increasing expectations for good cosmetic outcomes following breast surgery, the role of breast reconstruction with tissue expanders (TEs) has increased [1–6]. We previously reported the advantages of immediate breast reconstruction (IBR) using a skin flap with subcutaneous tissue [1]. This method is useful for covering the inferolateral region of the TE and provides a more expandable material for covering the inserted TE [1]. Our method shows a low rate of complications such as hematoma, skin necrosis, wound dehiscence, major infection, capsule contracture, and expander displacement [1, 6–8]. Given the low incidence of complications in IBR, the primary consideration for IBR patient selection is oncologic safety. Since less skin is resected in skin-sparing (SSM) or nipple-sparing mastectomy (NSM) compared to conventional mastectomy (Bt), it is feared that IBR with SSM or NSM could increase the risk of local recurrence [5, 9–14]. In our practice, we routinely check the closest distance between the tumor and dermis by preoperative ultrasound and appropriately resected the skin immediately overlying the tumor in cases when the tumor is located close to the skin. Using this procedure, we have yet to experience a skin flap recurrence. Of course, the influence of the tumor-to-dermis distance on local recurrence cannot be studied in a randomized controlled trial. However, this study reports the outcomes of IBR at our institution in terms of oncologic safety. Here, we compared the clinicopathological features of IBR using Bt with those of IBR using NSM/SSM. Preoperative ultrasound was used to assess the distance of the tumor to the dermis, rather than skin surface, in patients undergoing IBR; here, we evaluate the results in terms of oncological safety.

## Patients and methods

### Patients

This study enrolled 171 consecutive patients and 181 breasts with immediate reconstruction using an expander. All patients were treated at the Department of General Surgical Science, Graduate School of Medicine, Gunma University, from July 2009 to July 2019. Eighty-five breasts underwent skin-sparing or nipple-sparing mastectomy; 96 breasts underwent mastectomy. All patients included in this study gave their informed consent at the time of surgery for inclusion in future analyses. Patients whose tumors were attached to the skin or had invaded skin or muscle were excluded from the study. The details extracted from the database were age, histological type, primary tumor size (T factor), axillary lymph node status, estrogen receptor (ER) and progesterone receptor (PgR) expression status, the human epidermal growth factor receptor 2 (HER2) status of the primary tumor, and the use of adjuvant chemotherapy or hormone therapy. The ER and PgR statuses were assessed by the Allred score ≥3 indicating ER and PgR positivity [15, 16]. HER2 overexpression was determined by an immunohistochemistry (IHC) analysis and a fluorescence in situ hybridization (FISH) analysis with IHC 3+ or IHC 2+/FISH+ indicating HER2 positivity [16].

### Measurement of tumor distance from dermis (not skin surface)

**Fig. 1:**
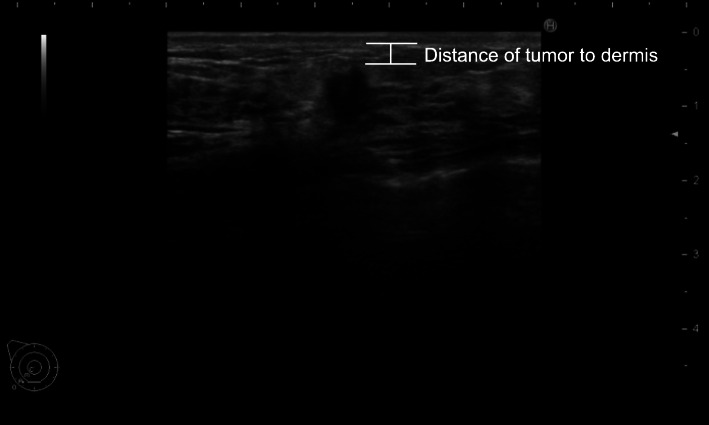
Representative cases. Tumor-to-dermis distance was measured perpendicularly in millimeters using the most anterior hypoechoic edge of the lesion. For tumors with indistinct margins, the boundary closest to the dermis was used to measure this distance

Breasts were routinely examined using an ultrasound (US) device (HI VISION Preirus ultrasonography system (Hitachi Ltd., Tokyo) with a 5–13 MHz linear array transducer (EUP-L74M)). All US images were digitally archived and subsequently retrieved for retrospective measurements of tumor proximity to the dermis. Tumor distance from the dermis was measured perpendicularly in millimeters from the dermis to the most anterior hypoechoic edge of the lesion (Fig. 1). In cases of tumors with an indistinct margin, the distance was measured based on the boundary closest to the dermis.

### Surgical procedure

Skin- or nipple-sparing mastectomy or total mastectomy was performed to remove the breast tissue under direct visualization [1, 5]. After completion of the mastectomy, dissection between the pectoralis major and pectoralis minor was performed. Then, the inferomedial attachments of the pectoralis major were detached as much as possible in order to secure adequate space and to prevent superior displacement of the TE. The expander was inserted into the space between the major and minor pectoral muscles, a partial subpectoral position for just superior pole coverage. Skin with subcutaneous tissue was used to cover the inferolateral portion of the expander for soft tissue support in the inferior pole. One suction drain was placed on the pectoralis major. Sentinel lymph node biopsy was performed with the radioactive dye method. In cases that were node-positive, axillary dissection was routinely performed.

### Statistical analysis

Breasts were separated into two groups for analysis: the 86 breasts that underwent skin-sparing or nipple-sparing mastectomy and the 96 breasts that underwent traditional mastectomy. We conducted a univariate statistical analysis using Fisher’s exact test or the *χ*^2^ test with Yates’s correction. To compare the two groups, we used Student’s *t*-test. Differences were considered statistically significant when *p*<0.05.

## Results

**Fig. 2:**
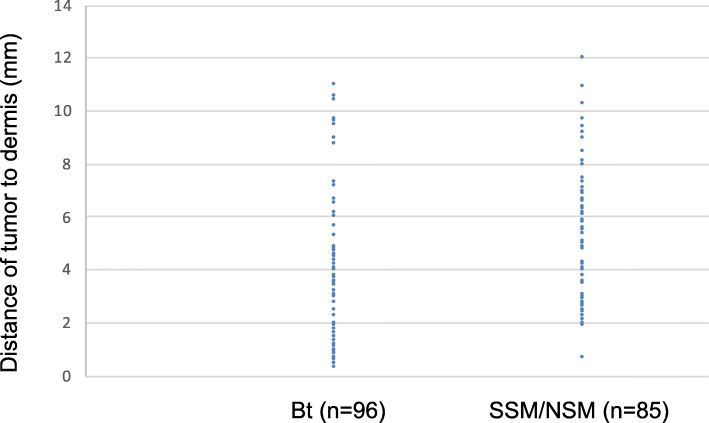
Tumor-to-dermis distance of cases with skin preservation (NSM/SSM) was significantly less than that of cases with skin resection (Bt) (3.8 ± 2.7 mm vs 5.2 ± 2.4 mm). By contrast, in cases with invasive carcinoma, 28 out of 78 cases in which Bt was performed (35.9%) had a tumor-to-dermis less than 2 mm, whereas 3 of the 63 cases upon which NSM/SSM was performed (4.8%) had a tumor-to-dermis distance less than 2 mm. In the 3 cases in which the tumor-to-dermis distance was less than 2 mm, the skin immediately overlying the tumor was resected

**Fig. 3:**
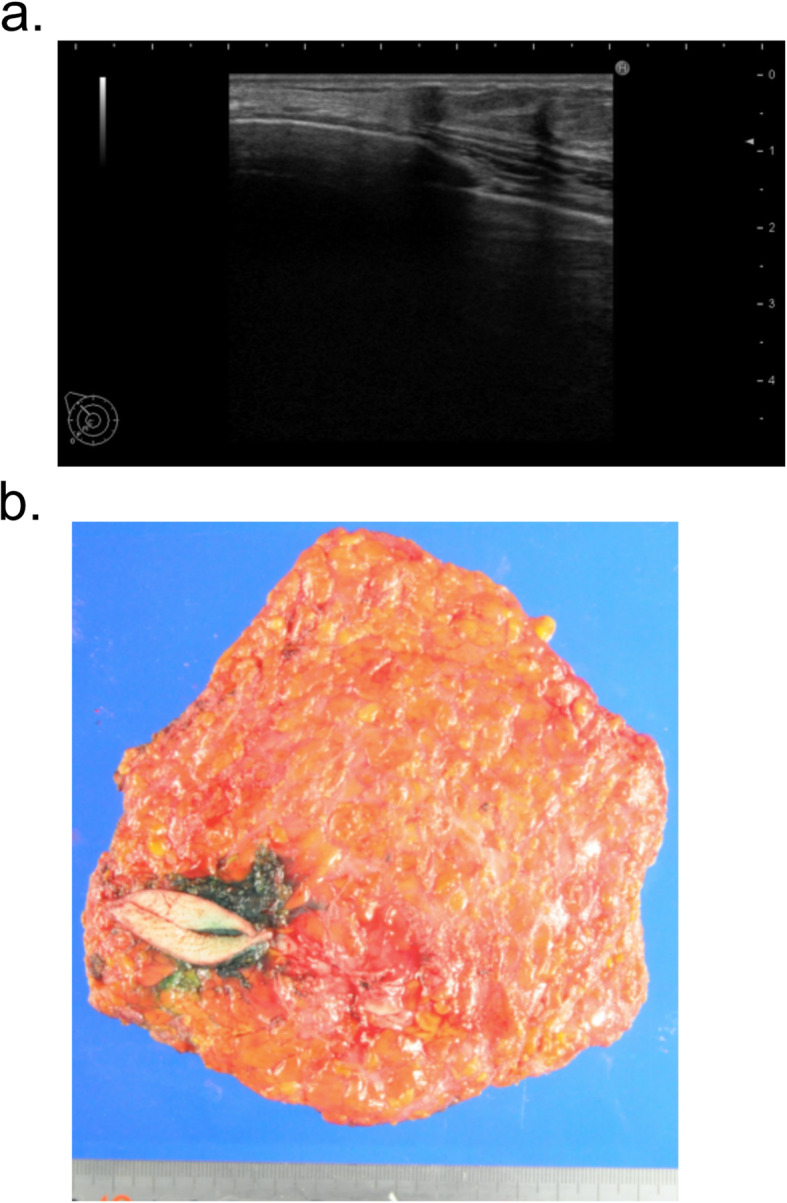
A case in which the tumor-to-dermis distance was less than 2 mm underwent resection of the skin immediately overlying the tumor. **a** Ultrasonography revealed that tumor-to-dermis distance was less than 2 mm. **b** Resection of the skin just above the tumor was performed

We analyzed the cases of 171 patients and 181 breasts with breast cancer that had undergone immediate two-stage breast reconstruction with an expander. The resected margins were all clear, and none of the patients required postoperative radiotherapy. The median age of the patients was 47 years with a range of 25–75 years. The overall median follow-up period was 47.1 months (range, 8.8–125.3 months). Among the total 181 reconstructed breast mounds, the locoregional recurrence rate was 1.1% (2 breasts) with no cases of skin flap recurrence. Distant recurrence was observed in 2 cases (1.2%). Table 1 compares the patient characteristics of the two groups, NSM/SSM and Bt, and summarizes the results of the univariate analysis. As can be seen, none of the clinicopathological features, including primary tumor progression, method of reconstruction, or use of adjuvant chemotherapy, were significantly different between the two groups. However, the distance of the tumor to dermis of cases with skin preservation (NSM/SSM) was significantly less than that of cases with skin resection (Bt) (3.8 ± 2.7 mm vs 5.2 ± 2.4 mm) (Table 1, Fig. 2). By contrast, in cases with invasive carcinoma, 28 cases out of 78 cases upon which Bt was performed (35.9%) had a tumor-to-dermis distance less than 2 mm, whereas only 3 of the 63 cases upon which NSM/SSM was performed (4.8%) had a tumor-to-dermis distance less than 2 mm. In the 3 cases in whom the tumor-to-dermis distance was less than 2 mm, the skin immediately overlying the tumor was resected (Figs. 2 and 3). Thus, in all cases in this study with a tumor-to-dermis distance less than 2 mm, the skin immediately overlying the tumor was resected. In cases with ductal carcinoma in situ (DCIS), 5 of the 18 cases upon which Bt was performed (27.8%) had a tumor-to-dermis distance less than 2 mm, whereas 3 of the 22 cases upon which NSM/SSM was performed (13.6%) had a tumor-to-dermis distance less than 2 mm. These patients with DCIS also had no recurrent disease.

**Table 1:**
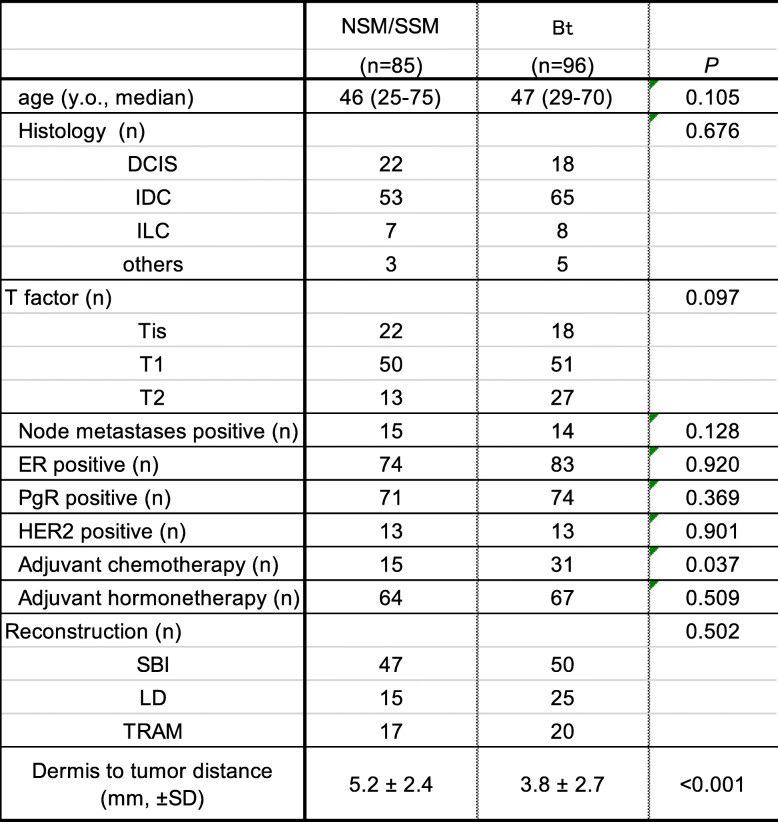
Patient characteristics and clinicopathological features associated with surgical procedure

Values are expressed as mean ±SD. *n* Number; *DCIS* Ductal carcinoma in situ; *IDC* Invasive ductal carcinoma; *ILC* Invasive lobular carcinoma; *SBI* Silicon breast implant

## Discussion

Surgery is still important as a primary treatment for breast cancer. Immediate breast reconstruction (IBR) with a tissue expander has become an increasingly popular procedure [1–6]. However, in the reported literature, the locoregional recurrence after SSM and NSM has been as high as 14.3% [5, 9–14] and is associated with distant metastasis and poor prognosis. Thus, locoregional recurrence is arguably the most important concern regarding IBR procedures. The key observations made in the present study are as follows: (1) Among a total of 181 reconstructed breast mounds, the locoregional recurrence rate was 1.1% (2 breasts), with no skin flap recurrence in patients undergoing IBR, and (2) in cases with invasive carcinoma, all cases in which the tumor-to-dermis distance was less than 2 mm underwent resection of the skin immediately overlying the tumor. Our results suggested that a tumor-to-dermis distance of at least 2 mm is adequate for local control of the skin flap in SSM/NSM procedures.

Preoperative evaluation of the distance between the skin and the tumor is critical for evaluation of whether SSM/NSM is possible. Breasts in our series were routinely examined using an ultrasound device. Previous studies have investigated the distance between the edge of the tumor and the skin surface in breast cancer patients [14, 17]. In this study, we focused on the distance between the tumor and the dermis. Given our results, we estimate that at least a tumor-to-dermis distance of at least 2 mm is needed to avoid local recurrence of the skin flap. In addition, the skin flap should be kept as thick as possible to preserve blood flow and avoid skin flap necrosis [1]. Fortunately, the lymphovascular network in the dermis is rich. In the current study, we had no cases of skin flap necrosis due to loss of blood flow. Thus, it appears that a tumor-to-dermis distance of at least 2 mm as measured by US is useful for avoiding skin flap necrosis, as well as tumor recurrence.

Ultrasound is not considered useful for evaluating the absolute distance between the edge of the tumor and the skin surface because it is necessary to press the probe onto the skin [14], and the underlying structures become deformed or displaced. However, in the context of tumor-to-dermis distance evaluation, it was effective in determining whether it is possible to preserve the skin. Furthermore, the US procedure has the advantage of being performed in the same supine position that the patient assumes during surgery, thus providing a relevant measure.

This study has potential limitations, the major one being that the number of cases was relatively small. In addition, we used retrospective methods of data collection. Additional research on a larger number of cases is needed to evaluate the clinical effectiveness of US evaluation and the appropriateness of the 2-mm tumor-to-dermis distance as a limit for SSM/NSM procedures. Unfortunately, this fundamental proposition cannot be studied in randomized controlled trials. To the best of our knowledge, this is the first report describing the significance of the tumor-to-dermis distance in IBR.

## Conclusions

In conclusion, we have demonstrated that among reconstructed breast mounds, we experienced no skin flap recurrence in patients who underwent IBR with SSM/NSM. Our results suggested that a 2-mm dermis-to-tumor distance by US evaluation is adequate for a skin flap in the procedure of SSM/NSM. We recommend that in cases with invasive breast cancer in which the dermis-to-tumor distance is less than 2 mm, the skin immediately overlying the tumor be resected.

## Data Availability

The datasets used and/or analyzed during the current study are available from the corresponding author on reasonable request.

## Abbreviations

DCIS: Ductal carcinoma in situ
ER: Estrogen receptor
FISH: Fluorescence in situ hybridization
HER2: Human epidermal growth factor receptor 2
IBR: Immediate breast reconstruction
IHC: Immunohistochemistry
NSM: Nipple-sparing mastectomy
PgR: Progesterone receptor
SSM: Skin-sparing mastectomy
TE: Tissue expander
US: Ultrasonography, ultrasound
